# Mucin 1 (Muc1) Deficiency in Female Mice Leads to Temporal Skeletal Changes During Aging

**DOI:** 10.1002/jbm4.10061

**Published:** 2018-07-14

**Authors:** Andrea M Brum, Cindy S van der Leije, Marijke Schreuders‐Koedam, Siham Chaibi, Johannes PTM van Leeuwen, Bram CJ van der Eerden

**Affiliations:** ^1^ Department of Internal Medicine Erasmus Medical Centre Rotterdam The Netherlands

**Keywords:** MUCIN 1, BONE, OSTEOBLAST, SKELETON, OSTEOCLAST

## Abstract

Mucin1 (MUC1) encodes a glycoprotein that has been demonstrated to have important roles in cell‐cell interactions, cell‐matrix interactions, cell signaling, modulating tumor progression and metastasis, and providing physical protection to cells against pathogens. In this study, we investigated the bone phenotype in female C57BL/6 *Muc1* null mice and the impact of the loss of *Muc1* on osteoblasts and osteoclasts. We found that deletion of *Muc1* results in reduced trabecular bone volume in 8‐week‐old mice compared with wild‐type controls, but the trabecular bone volume fraction normalizes with increasing age. In mature female mice (16 weeks old), *Muc1* deletion results in stiffer femoral bones with fewer osteoblasts lining the trabecular surface but increased endosteal mineralized surface and bone formation rate. The latter remains higher compared with wild‐type females at age 52 weeks. No difference was found in osteoclast numbers in vivo and in bone marrow osteoblast or osteoclast differentiation capacity or activity in vitro. Taken together, these results suggest that Muc1 depletion causes a transiently reduced trabecular bone mass phenotype in young mice, and later in life reduced numbers of osteoblasts with increased endocortical mineralization activity coincides with unaffected total bone mass and increased stiffness. In conclusion, our results show, for the first time to our knowledge, a role for Muc1 in bone mass and mineralization in mice in a time‐dependent manner. © 2018 The Authors. *JBMR Plus* published by Wiley Periodicals, Inc. on behalf of American Society for Bone and Mineral Research.

## Introduction

Bone is a dynamic organ, undergoing constant remodeling that is a balancing act between removal of old bone by osteoclasts and formation of new bone controlled by osteoblasts, as well as its terminally differentiated form, the osteocyte. A number of key factors and signaling pathways controlling osteoblast differentiation and activity have been identified over the last few decades. Runt‐related transcription factor 2 (Runx2) and osterix (Osx) have been identified to be two essential transcription factors required for osteoblast differentiation,^1^, ^2^, ^3^ and Wnt, bone morphogenetic protein (BMP), hedgehog (Hh), Notch, and transforming growth factor‐beta (TGFβ) are key signaling pathways in osteoblasts.^4^, ^5^, ^6^, ^7^, ^8^, ^9^ Still, a better understanding of the signaling network, their intricate interactions, and what other genes and pathways are involved in osteoblast function is required to develop approaches to enhance bone formation and promote fracture healing for patients with disorders such as osteoporosis and nonunion fractures. Gene expression analysis of differentiating osteoblasts showed an increase in Mucin 1 (*MUC1*) expression compared with undifferentiated human mesenchymal stromal cells (hMSCs),^10^ indicating a potential role for Mucin 1 in the dynamic bone formation and remodeling process.

Mucins were first identified as being a major component of many types of mucus and derive their name from the Greek word for slimy. These glycoproteins are high in O‐linked glycosylation sites, creating a dense, viscous, negatively charged molecule. To date, 21 different mucins have been identified (MUC1‐21) and can be divided into two groups: membrane‐bound mucins and secretory mucins. Mucin 1 (MUC1) was the first mucin cloned and sequenced,^11^, ^12^, ^13^, ^14^ and it encodes a high molecular–weight glycoprotein well known to be expressed on the apical surface of many epithelial cells. MUC1, like all other membrane‐bound mucins, is a type 1 membrane protein and is composed of an N‐terminal protein sequence, followed by a sequence encoding a variable number of tandem repeats (VNTR), then a transmembrane domain, and finally a cytoplasmic tail.^15^ This membrane‐bound protein can be released from the cell through proteolytic cleavage and, as a result of alternative splicing, a number of isoforms exist, including versions that lack a transmembrane domain altogether.^(15,16)^ MUC1 falls into a family of structurally related mucin‐like glycoproteins, including CD34, CD43, CD162, CD164, GlyCAM1, and MAdCAM1. MUC1 has been reported to play an important role in cell‐cell interactions, cell‐matrix interactions, modulating tumor progression and metastasis,^15^, ^16^, ^17^, ^18^, ^19^ and also serves as an adhesion molecule for a number of pathogens, providing cells protection from bacteria.^18^, ^20^, ^21^ The cytoplasmic tail of MUC1 functions in cell signaling, with seven evolutionary conserved tyrosine phosphorylation sites and cytoplasmic domains that interact with other key signaling molecules, including ß‐catenin, p53, and NF‐κB.^22^, ^23^

This study aimed to examine the skeletal phenotype of mice with global deletion of *Muc1*. To understand the dynamic effect *Muc1* deficiency may have on the skeleton over time, we scrutinized bone microarchitecture, strength, mineralization, and osteoblast and osteoclast differentiation and activity at several time points during aging.

## Materials and Methods

### Mice

*Muc1* ^−/−^ mice were constructed as described by Spicer and colleagues.^24^ The mice were backcrossed onto C56BL/6 and then wild‐type (WT) and *Muc1* ^−/−^ mice were bred from heterozygous parents in the same facility. Mice were genotyped by PCR using specific primer sets as follows: Wild‐type allele was detected using 5′‐ACC TCA CAC ACG GAG CGC CAG‐3′ and 5′‐TCC CCC CTG GCA CAT ACT GGG‐3′, and the mutant allele was detected by 5′‐ACC TCA CAC ACG GAG CGC CAG‐3′ and 5′‐CAA CTG TTG GGA AGG GCG AT‐3′. An amplified fragment of 260 bp corresponds to the WT allele and of 240 bp to the mutant allele.

Mice were housed in standard microisolator or individually ventilated cages in groups of 1 to 5 animals in specific pathogen‐free (SPF) rooms at a 12‐hour light/dark cycle and had *ad libitum* access to food and water. Female mice (*n* = 9–10 per genotype) were euthanized at 8, 16, and 52 weeks of age and bones were collected for microcomputed tomography and histomorphometry (left femurs), 3‐point bending tests (right femurs), and bone marrow cultures (tibias). Male mice (*n* = 9–10 per genotype) were euthanized at 8, 16, and 52 weeks of age and bones were collected for microcomputed tomography (left femurs) and 3‐point bending tests (right femurs). Mice were healthy for the duration of the study, with the exception of one WT female mouse in the longitudinal study/52‐week group that was found to have a wound at age 36 weeks (it was deemed too severe to survive and she was humanely euthanized) and one KO male mouse in the longitudinal study/52‐week group that was found dead at age 44 weeks without any apparent cause. To determine the number of mice used in each treatment group, power calculations were performed utilizing data from a pilot experiment assessing bone microarchitecture and strength parameters of bones from *Muc1* ^−/−^ and WT littermates provided by the Gendler Lab group. Researchers were blinded to the genotype of the mice during all experimental procedures and at death. All animal experiments were performed in compliance with the animal ethics board of the Erasmus Medical Center.

### Micro‐computed tomography (µCT)

In vivo µCT of the left femur was carried out on WT and *Muc1* ^−/−^ mice (*n* = 10) every 2 months from 3 months until age 11 months. The scans were performed at 20‐µm resolution using a Quantum FX system (PerkinElmer, Groningen, The Netherlands). Mice were anesthetized in an induction chamber by inhalation of 4% isoflurane. Under 4% isoflurane anesthesia, the animal was placed on a specially constructed holder that allows for fixation of the left femur. The holder with the animal was fixed on the animal bed of the scanner with a nose cone supplying 1.5% to 2.0% isoflurane in an air/oxygen mixture. The following scan settings were used: X‐ray power and tube current were 90 kV and 0.16 mA, respectively. Beam hardening and ring artifacts were reduced, and the total scan time was 3 minutes. For export of image files, the Quantum FX–generated VOX files were loaded into Analyze 12 software (AnalyzeDirect, Overland Park, KS, USA) where a cut‐off intensity of 900 to 2600 (or max if less than 2600) was applied and the complete set of multiple 2D images were saved in BMP format for downstream analysis. Analysis only includes data for 9 WT females at 38‐ and 47‐week time points because of the euthanization of one mouse for a wound as described previously. During the analysis of the in vivo scans, one scan from a KO female at the 47‐week time point was found to have motion blurring and was excluded from the analysis.

For ex vivo µCT analysis, the left femurs from female WT and *Muc1* ^−/−^ mice euthanized at 8, 16, and 52 weeks (*n* = 8–10) were scanned at a resolution of 8.82 µm, using a SkyScan 1076 system (Bruker MicroCT, Kontich, Belgium). According to guidelines recently published,^25^ the following settings were used: X‐ray power and tube current were 40 kV and 0.25 mA, respectively. Beam hardening (20%) was reduced using a 1‐mm aluminum filter, ring artifacts were reduced (set at 5), exposure time was 5.9 seconds, and an average of three pictures was taken at each angle (0.8°) to generate final images. 3D images were reconstructed using NRecon from Bruker MicroCT (http://www.skyscan.be/products/downloads.htm).

For both in vivo and ex vivo µCT data analysis, we used software packages from Bruker MicroCT (CtAn and Dataviewer). Bone microarchitectural parameters were assessed in cortical bone for the in vivo data and in both trabecular and cortical bone for the ex vivo data of all mice (*n* = 8–10 per genotype). For ex vivo scans (100 sections), the metaphyseal area was selected for analysis starting 135 (8‐week‐old mice), 110 (16‐week‐old mice), and 95 (52‐week‐old mice) sections below our offset landmark within the epiphyseal growth plate. The diaphyseal (50 sections for 8.82 µm ex vivo scans, 25 sections for 20 µm in vivo scans) area was selected for the midshaft of the femur. The trabecular bone parameters trabecular tissue volume, bone volume, trabecular volume fraction (BV/TV), trabecular thickness, trabecular number, and trabecular patterning factor (connectivity of trabeculae) were determined in the distal metaphysis of the femur. In the mid‐diaphysis, cortical volume, endocortical volume, cortical thickness, and perimeter were analyzed. For image processing, trabecular bone was manually selected, and cortical bone was automatically selected. Global thresholding was applied for segmentation using threshold levels of 85 (lower) and 255 (higher) for trabecular and levels of 140 and 255 for cortical bone measurements.

### Bone mechanical testing

Right femurs (*n* = 9–10) were stored in phosphate‐buffered saline at −20°C until further use. The procedure was carried out as previously described in detail.^26^ Briefly, femurs were placed in a custom‐made 3‐point bending device, with the loading posts 10 mm apart. Mechanical testing was performed, using a Single Column Lloyd LRX System (Lloyd Instruments, Fareham, UK). Displacement (mm) and force (N) were registered. From the resulting displacement to force graphs, ultimate force (N), stiffness (N/mm), and work to failure (mJ) were determined as described before.^27^

### Bone histomorphometry

To determine the dynamic histomorphometric indices, WT and *Muc1* ^−/−^ mice (*n* = 8–10) were injected with calcein (15 mg/kg, Sigma, St. Louis, MO, USA) intraperitoneally before euthanization on the following schedule: 5 and 2 days prior for 8‐week‐old mice, 6 and 2 days prior for 16‐week‐old mice, and 8 and 2 days prior for 52‐week‐old mice. After excision, femurs were routinely embedded in methyl methacrylate as described previously.^28^ For bone histomorphometry analysis, the blocks were sectioned at 6 µm. For osteoblast measurements, sections were stained with a Goldner staining as described before.^29^, ^30^ For detection of osteoclasts, tartrate‐resistant acid phosphatase (TRAP) staining was performed. Sections were rinsed in 0.2 M sodium acetate/100 mM tartaric acid for 20 minutes, after which Naphtol AS‐MX (0.5 mg/mL) and Fast red TR salt (1.1 mg/mL) (both from Sigma) were added and incubated for 20 minutes at 37°C. Sections were counterstained with hematoxylin for 15 seconds, rinsed, air‐dried, and embedded in glycergel (Agilent, Amstelveen, The Netherlands). Dynamic trabecular and cortical analysis was performed separately. Cortical measurements were performed in the metaphysis between 0.5 mm and 2.5 mm below the growth plate. The trabecular measurements were performed in the entire bone marrow region from 0.5 mm to 1.5 mm below the growth plate. Labeled surfaces were hand‐drawn within Bioquant (version 7.20; Bioquant Image Analysis Corporation, Nashville, TN, USA) resulting in the following measurements: mineral surface (MS), bone surface (BS), tissue volume (TV), mineral apposition rate (MAR), and bone formation rate (BFR).

For TRAP and Goldner stainings, at least five images in the trabecular area were taken with a Zeiss Axiovert 200 MOT system (Carl Zeiss BV, Jena, Germany) at 20× magnification (0.5 mm to 2.5 mm below the growth plate). All quantitative measurements were performed on longitudinal sections that were taken from the anterior‐posterior middle of the bone. All measurements were performed in ImageJ (NIH, Bethesda, MD, USA, available online at https://imagej.nih.gov/ij/): osteoblasts, osteoclasts, osteoblast surface (contact area with the bone), osteoclast surface (contact surface with the bone), and bone perimeters were determined by eye based on staining and morphology (cuboidal cells on the bone surface). The number of osteoblasts and osteoclasts as well as the surfaces were hand‐drawn, and the resulting pixel measurements were calculated back to mm^2^. Histomorphometric indices were defined and calculated according to the American Society for Bone and Mineral Research (ASBMR) nomenclature,^31^ and analysis was performed blinded toward the genotypes.

### Bone marrow cultures

Bone marrow cells derived from WT and *Muc1* ^−/−^ mice (*n* = 7–8) were isolated and cultured toward osteoclasts and osteoblasts as described in detail.^28^, ^32^ After 7 days of culture, TRAP staining was used to stain for osteoclasts.^28^ Osteoclast‐generated resorption pits on calcium phosphate coating of Osteo Assay plates (Corning Life Sciences BV, Amsterdam, The Netherlands) were stained by von Kossa after 10 days of culture. Alkaline phosphatase (ALP) and alizarin red staining were performed on osteoblast cultures at days 10 and 21 of culture, respectively, as described earlier.^28^ Osteoclast number and the number of nuclei per cell were measured using the freely available ImageJ software (version 1.41; http://rsbweb.nih.gov/ij/). ALP‐positive colonies and resorption surface were measured using the software package Bioquant (version 7.20; Bioquant Image Analysis Corporation). To evaluate the mineralized nodule formation in vitro, cell/matrix layers were washed with PBS, fixed with 10% formaldehyde, and stained with alizarin red S (Sigma) solution. To quantify alizarin red S bound to mineralized nodules in the cultures, cultures were stained with alizarin red S, extensively rinsed with water, and extracted with 10% (w/v) acetic acid for 30 minutes at room temperature, after which the wells were scraped and contents added to Eppendorf tubes and vortexed for 30 seconds. Mineral oil was added to the mixture and incubated at 85°C for 10 minutes and then the tubes were placed on ice for 5 minutes. The tubes were centrifuged at 20,000*g* for 15 minutes, and 250 µL supernatant was pipetted into a clean tube and 100 µL 10% NaOH was added to neutralize the solution. The dye concentrations in the extracts were determined by absorbance at 540 nm using a Victor2 plate reader (PerkinElmer Life and Analytical Science, Boston, MA, USA). For each test, two to three technical replicates (bone marrow culture wells) from each mouse were averaged. Results are combined from two independent experiments (*n* = 3–4 mice/experiment).

### Statistical analysis

Values displayed are mean ± SD. The ex vivo μCT parameters, histomorphometric data, femur length, mechanical loading tests, and parameters of cell culture analyses were compared between genotypes for all age groups separately using two‐tailed unpaired Student *t* tests. For the in vivo μCT data, significances were calculated using the two‐way ANOVA with Bonferroni's multiple comparison post hoc test. All statistical analyses were performed using GraphPad Prism 6.0 (GraphPad, La Jolla, CA, USA). Any *p* values <0.05 were considered significant.

## Results

### During aging, femoral cortical bone is increased in WT and *Muc1* ^−/−^ female mice

**Figure 1:**
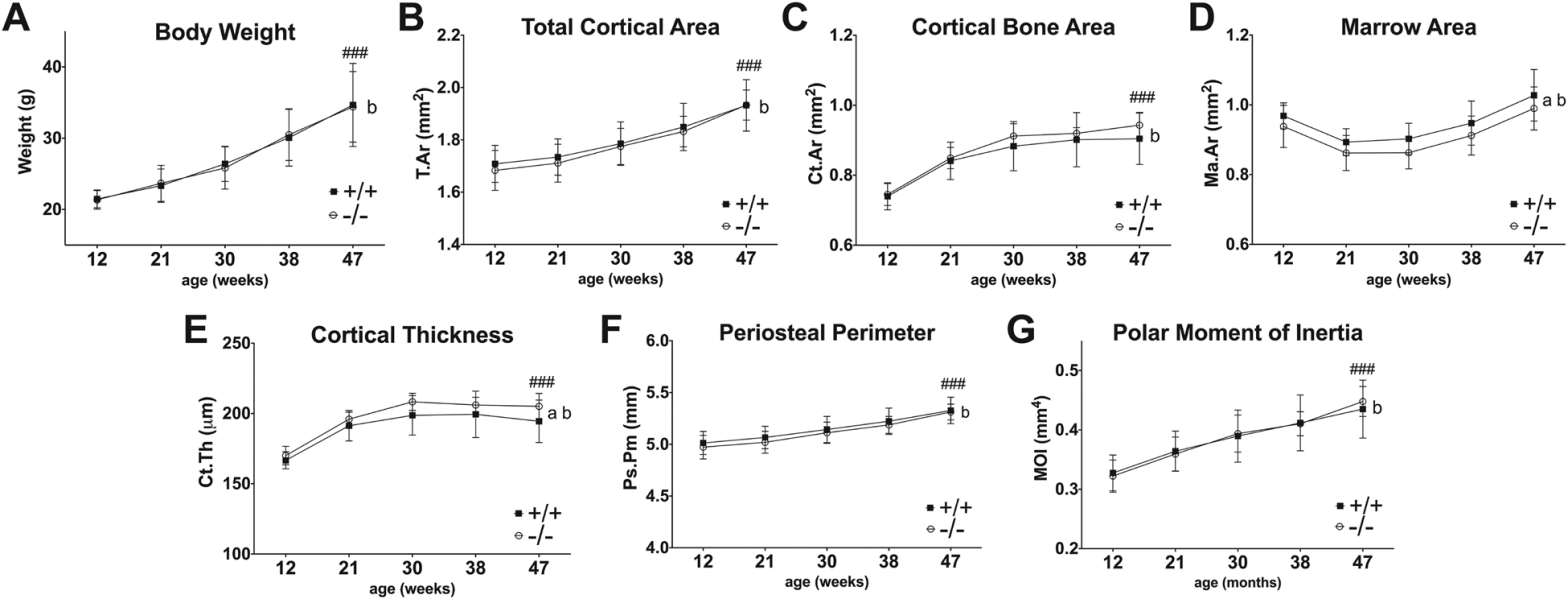
Longitudinal analysis of body weight and cortical bone structure in WT (+/+, filled squares) and *Muc1*‐deficient (‐/‐, open circles) female mice. Total body weight from 12 weeks to 47 weeks of age (*A*). In vivo μCT analysis of total cortical area (*B*), cortical bone area (*C*), marrow area (*D*), cortical thickness (*E*), periosteal perimeter (midshaft circumference) (*F*), and polar moment of inertia (*G*) were measured at diaphyseal areas. *n *= 9–10. Statistics: 2‐way ANOVA; a, b = *p* < 0.01. a = significant difference between wild‐type and *Muc1* ^−/−^ with aging; b = significant difference with aging within a genotype. Bonferroni post hoc test: ### = *p* < 0.001 significant difference between 12 weeks and 47 weeks within genotype for both WT and KO mice.

Consistent with previous studies,^24^ all mice developed normally, with no obvious abnormalities or illnesses. Additionally, to help rule out the possibility of bone architecture being affected by changes in overall body weight, fat deposition, or muscle mass, we also quantified body weight (Fig. 1 *A*, Supplemental Fig. S1*A*), femoral and tibial muscle weight (Fig. S1*B*, *C*), and subcutaneous, scapular, and gonadal adipose weights (Fig. S1*D–F*) at various time points and found no difference between WT and KO mice for any of these parameters.

An initial study was performed into the longitudinal bone dynamics in WT and *Muc1* KO mice in order to gain a thorough view of the changes in the cortical bone over time. Female mice were analyzed every 2 months throughout adult life (12 weeks to 47 weeks) for cortical bone architecture in the femoral diaphysis by repeated in vivo μCT. Aging affected all cortical bone parameters in both genotypes (Fig. 1 *B–G*). Both WT and *Muc1* ^−/−^ mice gained cortical bone (Fig. 1 *C*) over time, with a 22% and 27% increase in bone from 12 weeks to 47 weeks of age in WT and KO mice, respectively. A similar trend was found for cortical thickness (Fig. 1 *E*) in both genotypes. An overall significant difference between WT and KO mice with aging was found on marrow area (*p* = 0.002) (Fig. 1 *D*), and cortical thickness (*p* = 0.001) (Fig. 1 *E*); however, *Muc1* deletion had no significant effect on cortical bone architecture at any specific time point (Fig. 1 *B–G*). No significant interaction between genotype and age was observed. For all parameters, no significant difference was observed between WT and KO mice from 12 weeks of age onward.

### Female *Muc1* ^−/−^ mice have temporally reduced trabecular bone mass at young age

**Figure 2:**
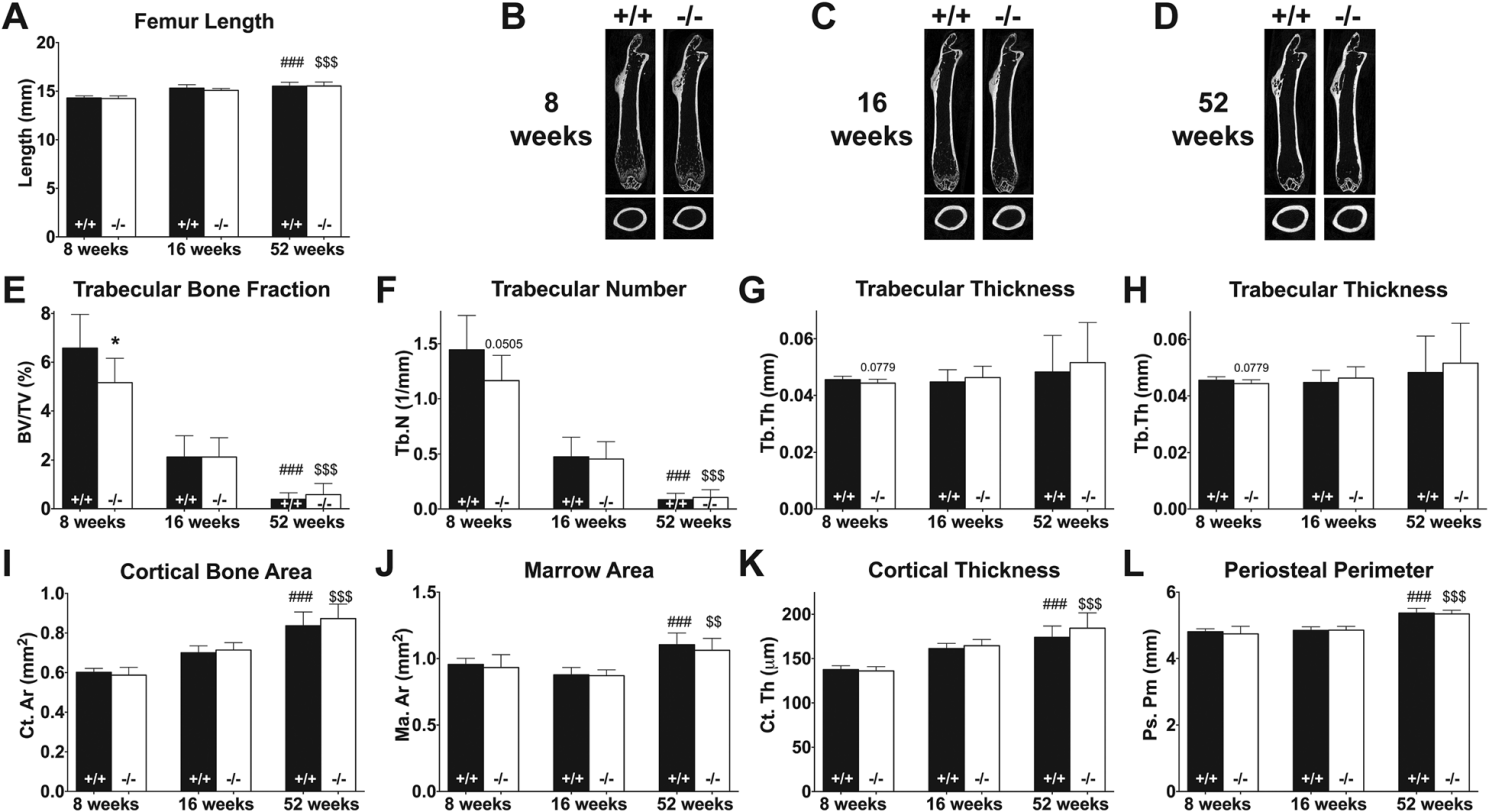
Ex vivo evaluation of macro‐ and microarchitecture of femoral bones from 8‐, 16‐, and 52‐week‐old WT (+/+, black bar) and *Muc1*‐deficient (−/−, white bar) female mice. Femur length of WT and KO mice (*A*). Representative longitudinal (top) and transverse (bottom) cross sections show bone shape and structure of WT (left) and *Muc1* ^−/−^ (right) mice at 8 weeks (*B*), 16 weeks (*C*), and 52 weeks (*D*) of age. Trabecular bone fraction (*E*), trabecular number (*F*), trabecular thickness (*G*), and trabecular separation (*H*) were measured at the metaphyseal region. Cortical bone area (*I*), marrow area (*J*), cortical thickness (*K*), and periosteal perimeter (midshaft circumference) (*K*) were measured at diaphyseal areas. For bone length, 8 weeks (*n* = 7 WT, *n* = 9 KO); 16 weeks (*n* = 7); 52 weeks (*n* = 8). For trabecular bone analysis, 8 weeks (*n* = 8 WT, *n* = 9 KO); 16 weeks (*n* = 9 WT, *n* = 10 KO); 52 weeks (*n* = 9 WT, *n* = 10 KO). For cortical bone analysis, 8 weeks (*n* = 8 WT, *n* = 9 KO); 16 weeks (*n* = 9 WT, *n* = 9 KO); 52 weeks (*n* = 9 WT, *n* = 10 KO). Statistics: Student *t* test exact *p* value and * = *p* < 0.05 WT versus *Muc1* ^−/−^ within time point. ### = *p* < 0.001 compared with 8‐week WT mice. $$ = *p* < 0.01 compared with 8‐week KO mice. $$$ = *p* < 0.001 compared with 8‐week KO mice.

To scrutinize trabecular, as well as cortical, femoral bone microarchitecture of WT and KO mice ex vivo, μCT analysis employing higher resolution (8.82 µm compared with 20 µm) was used at 3 time points (juvenile mice at 8 weeks, mature adult mice at 16 weeks, and aged mice at 52 weeks of age). For these more in‐depth analyses, the chosen time points included younger and older mice compared with the longitudinal study to expand the picture of the bone phenotype. The femur length (Fig. 2 *A*) and overall morphology (Fig. 2 *B–D*) of the bones did not differ between WT and *Muc1* knockout mice at any time point.

The trabecular bone volume fraction (BV/TV) in the metaphyseal region of 8‐week‐old KO mice was 22% lower (*p* = 0.028) than that of WT mice (Fig. 2 *E*), which can be primarily attributed to the strong trend found in lower number of trabeculae (19% of WT at 8 weeks, *p* = 0.051) (Fig. 2 *F*). No difference was found in trabecular thickness (Fig. 2 *G*), but trabecular separation was increased by 7% (*p* = 0.040) in KO mice compared with WT at age 8 weeks (Fig. 2 *H*). The difference in the metaphyseal phenotype between *Muc1* ^−/−^ and WT mice disappeared by age 16 weeks (Fig. 2 *E–H*) with an overall decrease of 88.8% (*p* < 0.001) and 94% (*p* < 0.001) in the trabecular bone fraction from 8 to 52 weeks in KO and WT mice, respectively. In the diaphyseal region, cortical parameters such as total cortical bone (Fig. 2 *I*), marrow area (Fig. 2 *J*), cortical thickness (Fig. 2 *K*), and periosteal perimeter (Fig. 2L) exhibited no difference between WT and KO mice at 8, 16, or 52 weeks of age. As was demonstrated by the in vivo μCT study, cortical bone increased with age, with a 39% increase (*p* < 0.001) in WT and 48% increase (*p* < 0.001) in KO mice for total cortical bone.

*Muc1* KO male mice show no difference, compared with WTs, in body weight (Fig. S2*A*) or femur length (Fig. S2*B*) at any time point, nor did they display a difference in any trabecular or cortical bone parameter at 8 and 16 weeks (Fig. S2*C–J*). At age 52 weeks, male KO mice display a mild reduction in the number of trabeculae (Fig. S2*D*) and increase in trabecular separation (Fig. S2*F*). Trends with aging for the male mice were similar to the females, with the exception that the male mice showed an increase in trabecular thickness (WT: 23%, *p* = 0.0013; KO: 24%, *p *= 0.0052) and separation (WT: 87%, *p* < 0.0001; KO: 133%, *p* < 0.001) at age 52 weeks compared with age 8 weeks. In addition, male KO mice displayed no difference in cortical thickness (*p* = 0.61) between 8 and 52 weeks of age, and WT males had a less substantial increase in cortical thickness (10%, *p* = 0.0498) between 8 and 52 weeks, which result in lower increases in total cortical bone area (WT: 19%, *p* = 0.0108; KO: 11%, *p* = 0.0182) as well.

### Sixteen‐week‐old *Muc1* ^−/−^ female mice have fewer trabecular osteoblasts, but endocortical bone formation rate is increased in 16‐ and 52‐week‐old *Muc1* ^−/−^ mice

**Figure 3:**
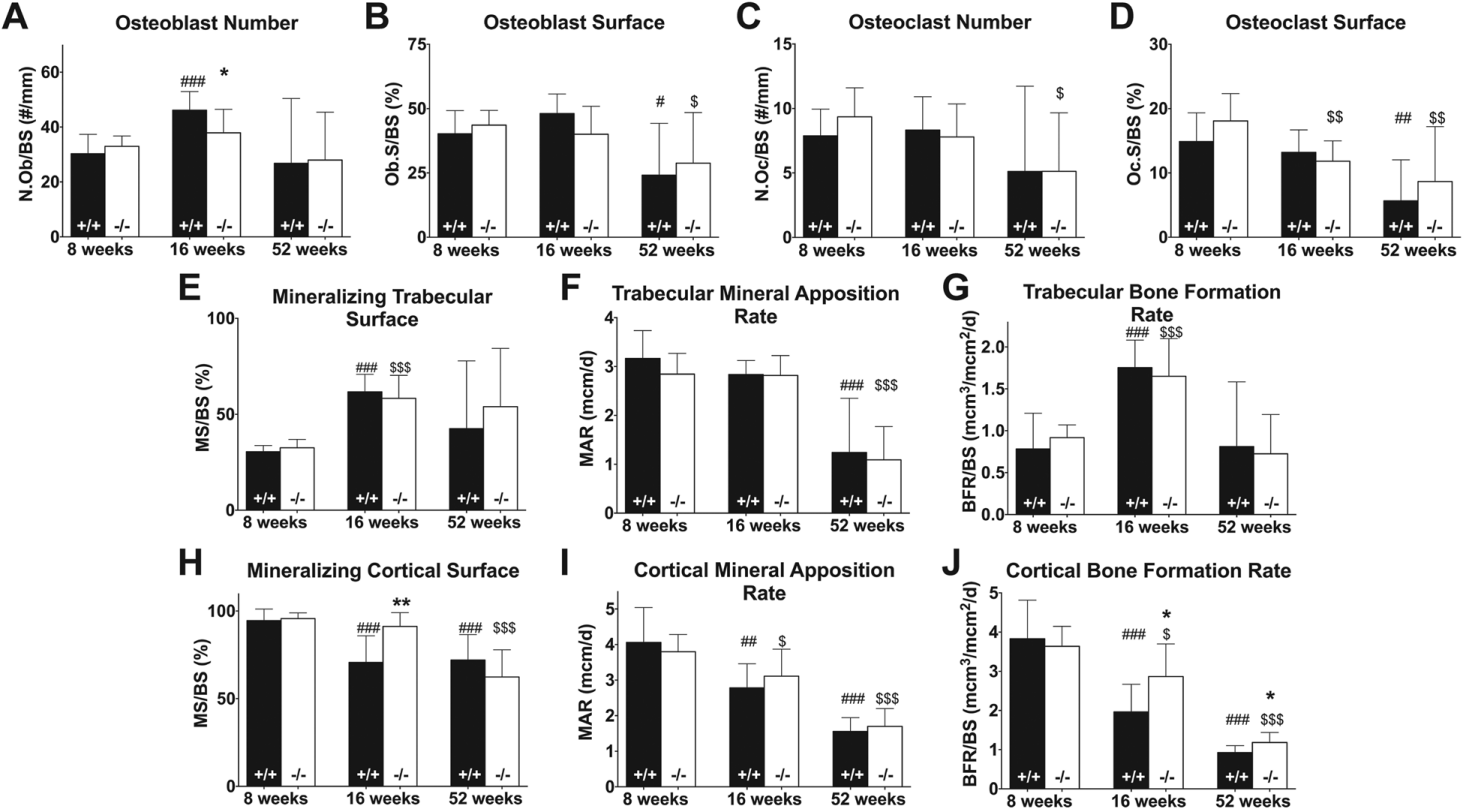
Static and dynamic histomorphometry of osteoblast and osteoclast number and osteoblast activity in femoral bone sections of WT (+/+, black bar) and *Muc1*‐deficient (−/−, white bar) female mice at 8, 16, and 52 weeks of age. Goldner stainings were performed to sections and images were taken of the metaphyses from which we quantified osteoblast number (*A*) and osteoblast covered surface (*B*). TRAP stainings were performed on femoral bone sections from WT (black bars) and *Muc1* ^−/−^ (white bars) mice and images were taken of the metaphyses. From these stainings, osteoclast number (*C*) and osteoclast surface (*D*) per bone surface area were quantified. Mice were injected with calcein before euthanization to label mineralization sites and allow for quantitative analyses of trabecular bone mineralizing surface (*E*), mineral apposition rate (*F*), and bone formation rate (*G*) and of cortical bone endosteal mineralizing surface (*H*), mineral apposition rate (*I*), and bone formation rate (*J*). Statistics: Student *t* test * = *p* < 0.05 WT versus *Muc1* ^−/−^ within time point. ** = *p* < 0.01 WT versus *Muc1* ^−/−^ within time point. # = *p* < 0.05 compared with 8‐week WT mice. ## = *p* < 0.01 compared with 8‐week WT mice. ### = *p* < 0.001 compared with 8‐week WT mice. $ = *p* < 0.05 compared with 8‐week KO mice. $$$ = *p* < 0.001 compared with 8‐week KO mice. For osteoblast bone analysis, 8 weeks (*n* = 10 WT, *n* = 9 KO); 16 weeks (*n* = 9 WT, *n* = 10 KO); 52 weeks (*n* = 9 WT, *n* = 9 KO). For osteoclast bone analysis and trabecular bone calcein analysis, 8 weeks (*n* = 10 WT, *n* = 9 KO); 16 weeks (*n* = 9 WT, *n* = 10 KO); 52 weeks (*n* = 9 WT, *n* = 10 KO). For cortical calcein analysis, 8 weeks (*n* = 8 WT, *n* = 9 KO); 16 weeks (*n* = 9 WT, *n* = 10 KO); 52 weeks (*n* = 9 WT, *n* = 10 KO).

Histological sections of femurs from female WT and *Muc1* ^−/−^ mice were stained with Goldner trichrome to assess the number and distribution of cuboidal osteoblasts or with TRAP to assess the number and distribution of osteoclasts in the bone. We found that the number of osteoblasts in the femurs of 16‐week‐old *Muc1* ^−/−^ mice was 18% lower (*p* = 0.034) compared with WT mice, but the numbers did not differ in 8‐ or 52‐week‐old mice (Fig. 3 *A*). Although a trend was observed in percentage of osteoblast‐covered surface between WT and KO mice at 16 weeks of age (*p* = 0.0809), no significant difference was observed between WT and KO mice at any time point (Fig. 3 *B*). Histological analysis of TRAP‐stained femoral sections showed no difference in the number of osteoclasts or the percent of bone covered by osteoclasts at any time point between the bones of WT and *Muc1* ^−/−^ mice (Fig. 6 *C*, *D*). A decrease in the percent of bone surface covered by osteoblasts (Fig. 3 *B*) (WT, *p* = 0.036; K, *p* = 0.046) and osteoclasts (Fig. 3 *D*) (WT, *p* = 0.002; KO, *p* = 0.008) between 8‐week‐old and 52‐week‐old mice was observed for both WT and KO groups. The relative number of osteoclasts on the femoral bone surface decreased only in KO mice between 8 weeks and 52 weeks of age (Fig. 3 *C*) (WT, *p* = 0.225; KO, *p* = 0.022).

To determine if *Muc1* deletion affects mineralization and bone formation rates, dynamic histomorphometric analysis was performed in sections of femurs from female WT and KO mice injected twice with calcein before they were euthanized. Trabecular mineralizing surface fractions, mineral apposition rates, and bone formation rates did not differ between WT and KO mice (Fig. 3 *E–G*). In both groups of mice, trabecular mineral apposition rates decreased over time, whereas bone formation rates were highest at age 16 weeks, nearly double that of rates at 8 (WT, *p* < 0.001; KO, *p* < 0.001) and 52 weeks of age. The percentage of actively mineralizing endosteal cortical bone surface was 29% higher (*p* = 0.002) in 16‐week‐old *Muc1* ^−/−^ mice compared with WT (Fig. 3 *H*). Mineral apposition rate in cortical bone did not differ between WT and KO mice at any time point (Fig. 3 *I*), but bone formation rate in the cortex was significantly higher in KO mice at 16 (46% higher, *p* = 0.021) and 52 (28% higher, *p* = 0.022) weeks of age compared with WT controls (Fig. 3 *J*). For both parameters, a decline with increasing age was found, with the rate decreasing 76% (MAR, *p* < 0.001; BFR, *p* < 0.001) and 63% (MAR, *p* < 0.001; BFR, *p* < 0.001) between 8 and 52 weeks in WT and KO mice, respectively.

### Female *Muc1* knockout mice have stiffer bones at age 16 weeks

**Figure 4:**
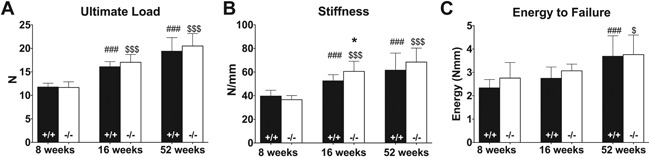
Mechanical testing of femurs of 8‐, 16‐, and 52‐week old WT (+/+, black bar) and *Muc1*‐deficient (−/−, white bar) female mice. Three‐point bending tests performed on the femurs of WT and KO mice allowed for quantification of the energy to failure (*A*), ultimate load (*B*), and bone stiffness (*C*) of the bones. Statistics: Student *t* test * = *p* < 0.05 WT versus *Muc1* ^−/−^ within time point. ### = *p* < 0.001 compared with 8‐week WT mice. $ = *p* < 0.05 compared with 8‐week KO mice. $$$ = *p* < 0.001 compared with 8‐week KO mice. 8 weeks (*n* = 9 WT, *n* = 9 KO); 16 weeks (*n* = 9 WT, *n* = 10 KO); 52 weeks (*n* = 9 WT, *n* = 10 KO).

Structural and compositional changes in the bone can lead to changes in bone strength. To determine if the strength of the femurs was affected in mice lacking *Muc1*, we performed 3‐point bending tests and measured three parameters: energy to failure, ultimate load, and stiffness.^33^ In female mice, no difference was found for ultimate load (Fig. 4 *A*), but bone stiffness was increased by 15% (*p* = 0.028) in 16‐week‐old KO compared with WT mice (Fig. 4 *B*). Energy to failure (Fig. 4 *C*) did not differ between WT and KO mice at any time point.

As with female mice, male KO and WT mice did not differ in ultimate load (Supplemental Fig. S3*A*) or energy to failure (Supplemental Fig. S3*C*), but male KO mice did display stiffer femoral bones at 8 weeks of age compared with WT males (21%, *p* = 0.0303); however, this difference disappeared by 16 weeks of age (Supplemental Fig. S3*B*).

### Bone marrow‐derived osteoblast differentiation does not differ between WT and KO mice

**Figure 5:**
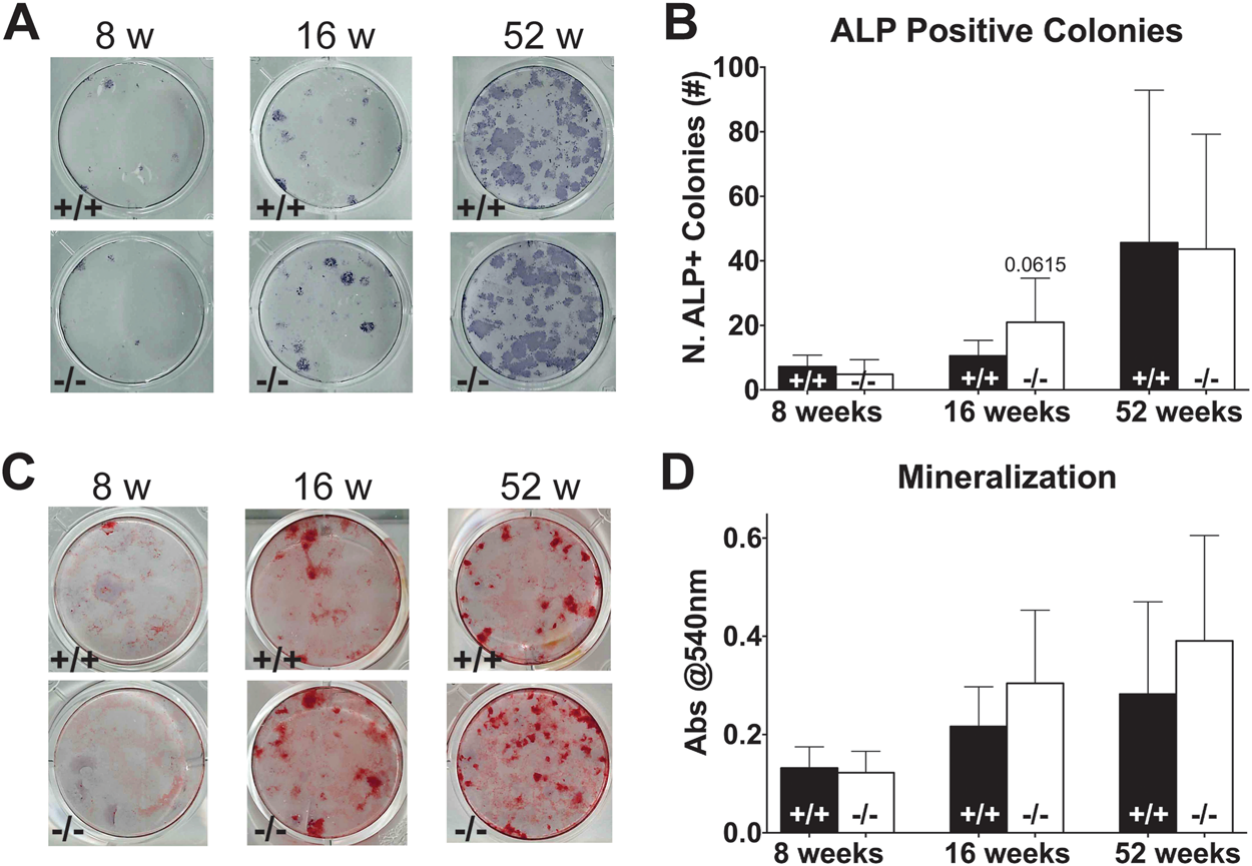
Osteoblast cultures of bone marrow from 8‐, 16‐, and 52‐week‐old WT (+/+, black bars) and *Muc1*‐deficient (−/−, white bars) female mice. Analyses were performed of osteoblast differentiation capacity and activity in bone marrow cell cultures. Representative stainings of alkaline phosphatase (ALP)‐positive osteoblast colonies (*A*) in WT (top) and *Muc1−/−* (bottom) mice and quantification of the number of ALP‐positive colonies (*B*). Representative alizarin red stainings of osteoblast produced mineralization (*C*) in WT (top) and Muc1^−/−^ (bottom) mice and quantification of alizarin red staining indicative of mineralization activity of bone marrow–derived osteoblasts (*D*). Statistics: Student *t* test * = *p* < 0.05 WT versus *Muc1^−/−^*. For ALP analysis, 8 weeks (*n* = 8 WT, *n* = 7 KO); 16 weeks (*n* = 8); 52 weeks (*n* = 7 WT, *n* = 8 KO). For alizarin red analysis, 8 weeks (*n* = 8 WT, *n* = 7 KO); 16 weeks (*n* = 8); 52 weeks (*n* = 8). Results represent the combined results of 2 independent experiments.

To study the differentiation capacity of mesenchymal stromal cells (MSCs) toward osteoblasts, we isolated bone marrow containing MSCs and osteogenic progenitors from 8‐, 16‐, and 52‐week‐old female WT and *Muc1* ^−/−^ mice and analyzed their differentiation into osteoblasts in ex vivo cultures. After 1 week of culture, we counted the number of ALP‐positive colonies. No difference was observed in the number of colonies between marrow cultures from WT and KO mice, but a trend was found for an increased number of osteoblast colonies in bone marrow cells from 16‐week‐old KO mice (*p* = 0.062) (Fig. 5 *A*, *B*). After 3 weeks of culture in osteogenic differentiation medium, cells were stained with alizarin red to determine the level of mineralization in the bone marrow culture. No difference in mineralization from cells from WT and KO mice was observed (Fig. 5 *C*, *D*).

### Bone marrow‐derived osteoclast differentiation capacity and activity are not affected by depletion of *Muc1*

**Figure 6:**
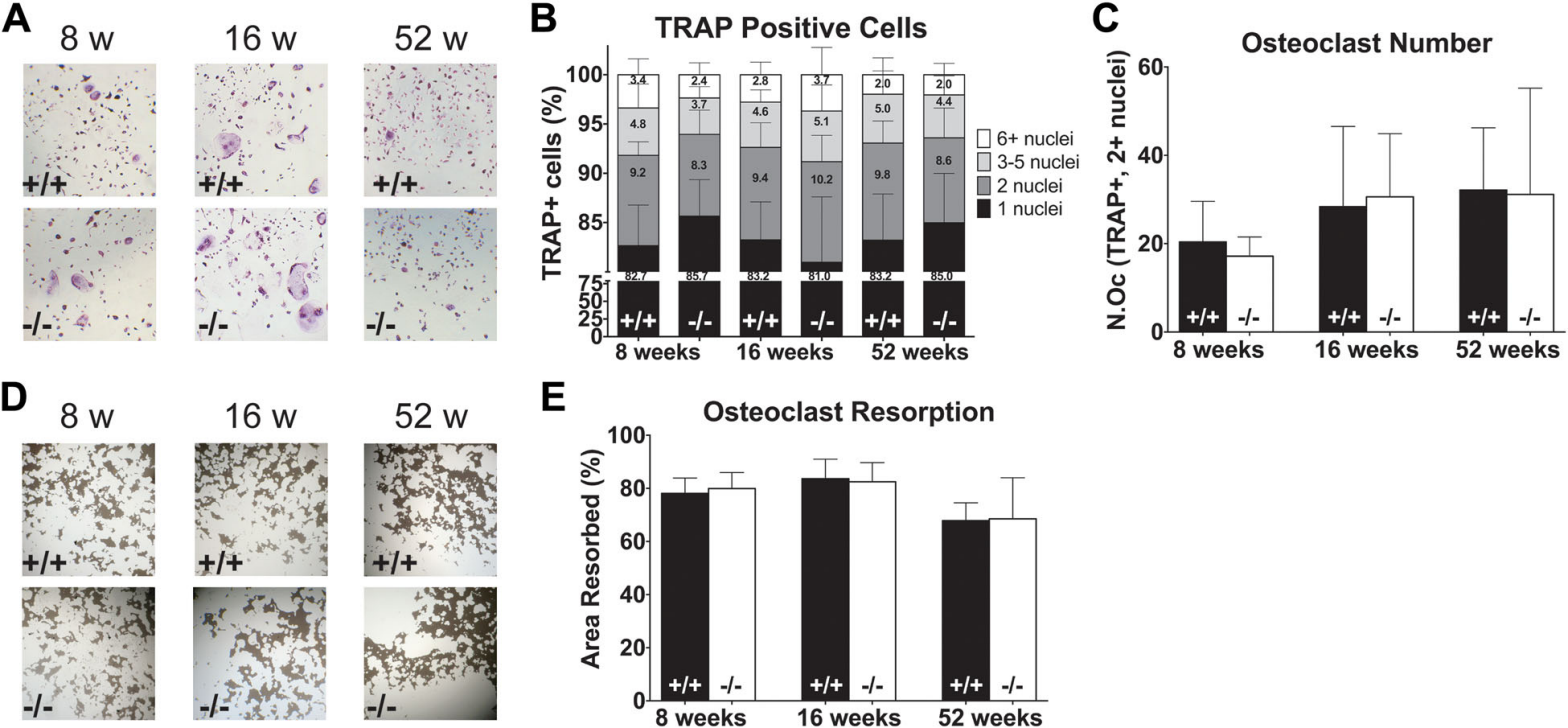
Osteoclast cultures of bone marrow cells from 8‐, 16‐, and 52‐week‐old WT (+/+) and *Muc1*‐deficient (−/−) female mice. Assessment of osteoclast differentiation capacity and activity in bone marrow cell cultures in WT and *Muc1* ^−/−^ mice was performed. Representative stainings of tartrate‐resistant acid phosphatase (TRAP)‐positive osteoclasts (*A*) in WT (top) and *Muc1^−/−^* (bottom) mice and quantification of the distribution in number of nuclei contained within TRAP‐positive osteoclasts (*B*) and the total number of multinucleated TRAP‐positive osteoclasts (*C*). Representative images of von Kossa staining showing resorption activity of osteoclasts (*D*) from bone marrow of WT (top) and *Muc1^−/−^* (bottom) mice and quantification of surface resorption by osteoclasts of WT and *Muc1* ^−/−^ mice (*E*). Statistics: Student *t* test * = *p* < 0.05 WT versus *Muc1* ^−/−^. 8 weeks (*n* = 8 WT, *n* = 7 KO); 16 weeks (*n* = 8); 52 weeks (*n* = 8). Results represent the combined results of 2 independent experiments.

Although our hypothesis is that *Muc1* plays a role in murine bone because of changes in osteoblast function, Leong and colleagues have found that human monocyte/macrophage cells express moderate levels of Muc1 protein,^34^ and we find that differentiating murine osteoclasts express low but detectable levels of *Muc1* (Supplemental Fig. S4). To study if *Muc1* deletion affects the differentiation capacity of PBMCs toward osteoclasts and/or osteoclast activity, we isolated bone marrow from 8‐, 16‐, and 52‐week‐old female WT and *Muc1* ^−/−^ mice and analyzed their differentiation into osteoclasts and their resorption capacity in ex vivo cultures. From the bone marrow samples, we saw no difference in the relative number of small or large TRAP‐positive osteoclasts or the total number of multinucleated osteoclasts (Fig. 6 *A–C*), nor did the resorption capacity of osteoclasts differ between cells from WT or KO mice (Fig. 6 *D*, *E*).

## Discussion

The results of this extensive and detailed skeletal phenotype analysis demonstrate that deletion of *Muc1* in mice leads to mild and temporally shifting changes in long bones and bone metabolism.

Eight‐week‐old female *Muc1* ^−/−^ mice display decreased trabecular bone compared with WT females; however, this difference disappears by 16 weeks. At the same time, endocortical bone formation rate and femoral stiffness are increased at 16 weeks of age in *Muc1*‐deficient female mice, with a higher rate of endocortical bone formation persisting to 52 weeks of age in KO mice. Histomorphometric analysis demonstrated that femurs of 16‐week‐old *Muc1* ^−/−^ female mice displayed lower numbers of osteoblasts lining the bone surface, whereas femurs from 8‐ and 52‐week‐old KO and WT mice did not differ in their number of osteoblasts, again demonstrating a temporal effect of *Muc1* deletion.

### Changes in murine femoral bone with aging

In both WT and *Muc1* ^−/−^, we observed a number of changes in the femoral bone over time. We found that the trabecular bone fraction dramatically declined between 8 and 52 weeks of age (88.8% in KO and 94% in WT mice). These rates of loss are similar to a previously published study looking at age‐related changes in the femoral bones of female wild‐type C57Bl/6 mice.^35^ We also saw that both WT and *Muc1* ^−/−^ mice gained cortical bone with aging (39% increase in WT and 48% increase in KO) between 8 and 52 weeks, and a similar trend was found for cortical thickness. Although Glatt and colleagues found that femoral cortical bone area remained steady between 2 and 12 months and cortical thickness in female C57Bl/6 mice increased markedly between 1 and 3 months of age and was then maintained through 12 months,^35^ our results showed an increase between each time point, although the gain is less between 16 and 52 weeks. The difference is likely to be explained by the method of analysis: whereas Glatt used the distal femur region to assess both trabecular and cortical bone properties, we used the midshaft of the femur for our cortical measurements. Consistent with the increase in cortical bone with aging, all mechanical strength properties increased with time as well. The larger, thicker cortex of the older bones directly contributes to a stronger femur, requiring more force to fracture, assuming the material properties are the same. In this study, trabecular bone formation rate peaked at 16 weeks, whereas cortical bone formation rate declined over time. In concordance, Glatt found that adult (5‐month‐old) female mice had a higher level of trabecular bone formation rate relative to younger (1‐ and 3‐month old) mice; however, they did not see a decrease later in life (12 months).^35^ Because the time points between the two studies are not identical, it is possible that this could be the reason for the observed difference. Cortical bone formation rate was not investigated in their study.^35^ The peak in trabecular bone formation rate at age 16 weeks goes along with the peak in osteoblast number that we also saw at 16 weeks of age.

### *Muc1*‐deficient mice display a temporally shifting bone phenotype

In 8‐week‐old *Muc1*‐deficient mice, trabecular bone volume fraction was reduced primarily because of lower trabecular number. Interestingly, although we observe a 22% decrease in the fraction of trabecular bone, no other bone parameter is altered in young mice, including trabecular mineralization rates, total number of osteoblasts, and bone strength parameters. This may indicate that osteoblast differentiation capacity and activity are not affected at 8 weeks of age and the reason for the lower amount of trabecular bone may lie in a preceding developmental defect or a hindrance in the growth plate in mice younger than the ages we studied. Another possibility is that lack of *Muc1* expression in osteoblasts could cause increased degradation of estrogen receptor alpha (ERα), which can lead to decreased bone mass. Previous studies have shown that the MUC1 C‐terminal subunit binds directly to the ERα DNA binding domain and stabilizes ERα by blocking its ubiquitination and degradation.^36^ Indeed, mice lacking ERα expression in osteoblasts display decreased trabecular and cortical bone mass in tibial, femoral, and vertebral bones.^37^ Unfortunately, they only studied mice at 12 and 18 weeks of age precluding observing temporal effects at ages we studied. Because we only see decreased trabecular bone in young *Muc1* ^−/−^ mice, it is possible that the temporal trabecular phenotype is overcome by redundancy in the system, for example, by other MUC proteins. Future studies demonstrating how estrogen deprivation may affect the phenotype in *Muc1* KO mice would greatly add to our understanding the role of *Muc1* in bone.

The bone phenotype of *Muc1* ^−/−^ mice changes over time. By 16 weeks of age, trabecular bone volume in *Muc1* ^−/−^ mice has normalized to the level of wild types, but cortical bone formation rate and stiffness are increased. As the mice age, even though the total number of osteoblasts in the bone is lower in KOs, they have an increased capacity to produce cortical bone, as illustrated by higher endocortical mineralized surface and mineral apposition rate at 16 and 52 weeks of age compared with WT mice. It has been previously established that stiffer bones result from increased cortical bone and/or changes in the material properties of the bone.^33^ The increase in stiffness in the KO mice is paralleled by increased cortical mineralizing surface and bone formation rate, which could lead to changes in the material properties of the bone and explain the increased stiffness. Future studies involving proteomic analysis of the bone matrix proteins in mice lacking *Muc1* would be useful in delineating the mechanism behind these changes in osteoblast activity and increased femoral stiffness. Two‐way ANOVA analysis showed an overall significant difference in marrow area and cortical thickness between WT and *Muc1* ^−/−^ with aging, but no significant effect was found at any one time point by post hoc or Student *t* test. The mild bone architectural phenotype we observed may be partially due to the mouse strain; C57Bl/6 mice are known to have a lower bone mass than other strains of mice, and this may limit observations on genetic changes in bone microarchitecture.

*Muc1* deletion also caused mild temporally shifting effects in the long bones of male mice; however, these effects differed somewhat from the females. *Muc1* ^−/−^ male mice show no difference, compared with WTs, in any trabecular or cortical bone parameter at 8 and 16 weeks. At 52 weeks of age, male *Muc1* ^−/−^ mice display a mild reduction in the number of trabeculae and increase in trabecular separation. These data are in contrast to the female *Muc1^−/−^* data in which we found that the trabecular bone fraction was strongly reduced at 8 weeks of age but no difference in trabecular bone parameter between *Muc1* ^−/−^ and WT mice later in life. Femoral stiffness was also temporarily increased in *Muc1* ^−/−^ male mice, but at 8 weeks of age, earlier than in females. Differences between males and females could partially be related to differences in estrogen regulation in males and females and regulation of ERα as discussed above. Further experiments are required to determine the mechanism of action of *Muc1* in the bones of both female and male mice; however, the male data support the overall conclusion that *Muc1* deletion has a temporal effect on bone biology.

### The importance of utilizing multiple time points when evaluating murine models for skeletal abnormalities

Our results demonstrating a temporal bone phenotype in *Muc1* ^−/−^ mice underpin the importance of looking at multiple time points when investigating in vivo bone phenotypes. Analyzing only a single time point in our study, as is common routine in many mouse genetic studies, would have led to either overinterpretation or missing aspects of *Muc1* deficiency on bone metabolism. We are not alone in identifying a transient or changing bone phenotype in genetically altered mice as many other studies report age‐dependent effects after gene perturbations. Mice genetically engineered to overexpress epidermal growth factor receptor ligand amphiregulin (AREG) demonstrate greater femoral BMD and total bone in the metaphyseal region at 4 and 8 weeks of age compared with WT controls, but after 10 weeks of age, there is no difference between the genotypes anymore.^38^ In addition, Shi and colleagues identified that deletion of BMP receptor BMPR1B resulted in trabecular bone loss at 8 weeks of age, which was not found either just after birth or at 11 weeks of age.^39^ Our lab also previously reported a temporal difference in skeletal changes with age between trabecular and cortical bone in DNA repair–deficient female trichothiodystrophy (TTD) mice where these mice show accelerated bone aging after 39 weeks of age.^40^ Taken together, our results suggest that the bone, and in particular the metaphysis of the long bones due to its relatively high bone turnover, may be most sensitive to gene/protein deficiency in an age‐related fashion, ie, coinciding with the time of most intense longitudinal growth and shows great temporal dynamics.

## Conclusion

In conclusion, this study demonstrates, for the first time to our knowledge, a role for *Muc1* in bone biology. Further studies are required to understand the mechanism of action by which *Muc1* affects osteoblast differentiation and bone formation in these mice and the reason for the temporal dynamics of the effects on bone. Given its explicit expression patterns in early human osteoblast differentiation,^10^ it will also be pivotal to investigate the expression patterns of *MUC1* in human MSCs and osteoblasts and its potential role in human bone homeostasis.

## Disclosures

All authors state that they have no conflicts of interest.

## Supporting information

Supporting Data S1.
