# Supplementary material for: Mucin 1 (Muc1) Deficiency in Female Mice Leads to Temporal Skeletal Changes During Aging
Source: JBMR Plus. 2018 Jul 14;2(6):341–50. doi: 10.1002/jbm4.10061 (PMC6237209; doi:10.1002/jbm4.10061)

**Supporting Information**

**SI Materials and Methods**

*Collection of Muscle and Adipose Tissues*

After sacrifice the musculus gastrocnemius of the tibia and the quadriceps muscle of the femur were dissected out from the left hindlimb and weighed immediately. Scapular adipose deposits (representative brown fat), subcutaneous adipose deposits (white adipose tissue), and gonadal adipose deposits (white adipose tissue) from the right side of each mouse were identified and excised as described by Mann et. al. [1]. Immediately after dissection the tissues were weighed. Weights are expressed as a percent of total body weight for each mouse.

*Quantification of mRNA expression*

RNA isolation, cDNA synthesis and PCR reactions were performed as described previously [2]. Oligonucleotide primer pairs were designed to be either on exon boundaries or spanning at least one intron (Table S5). Gene expressions were corrected for the housekeeping gene *Hprt*. Experiment was performed in triplicate.

**SI References**

[1] A. Mann, A. Thompson, N. Robbins, A.L. Blomkalns, Localization, identification, and excision of murine adipose depots., J. Vis. Exp. (2014). doi:10.3791/52174.

[2] C. Bruedigam, M. van Driel, M. Koedam, J. van de Peppel, B.C.J. van der Eerden, M. Eijken, J.P.T.M. van Leeuwen, Basic techniques in human mesenchymal stem cell cultures: differentiation into osteogenic and adipogenic lineages, genetic perturbations, and phenotypic analyses., Curr. Protoc. Stem Cell Biol. Chapter 1 (2011) Unit1H.3. doi:10.1002/9780470151808.sc01h03s17.

**SI Tables**

Table S5

| Gene | Forward 5'-3'' | Reverse 5'-3'' |
| --- | --- | --- |
| *Muc1* | CCCTATGAGGAGGTTTCGGC | GTGGGGTGACTTGCTCCTAC |
| *Hprt* | TTATCAGACTGAAGAGCTACTGTAATGATC | TTACCAGTGTCAATTATATCTTCAACAATC |

Oligonucleotide primers used in the study**.** Sequences of primer sets used for qPCR in this study. All genes were detected using SYBR green.

**SI Figure Legends**

Figure S1: Cross sectional study of body, muscle and adipose depot weights from WT (+/+, black bar) and *Muc1* deficient (-/-, white bar) mice at 8, 16, and 52 weeks of age. Total body weight (A). Femoral (B) and tibial (C) muscle weight relative to total body weight. Relative weights of subcutaneous (D), scapular (E), and gonadal (F) adipose deposits. Statistics: student t-test * = p<0.05 WT vs. Muc1^-/-^ comparing WT versus KO at each time point. For body weight 8 weeks (n=10 WT, n=9 KO); 16 weeks (n=9 WT, n=10 KO); 52 weeks (n=9 WT, n=10 KO). For muscle weights 8 weeks (n=10 WT, n=9 KO); 52 weeks (n=8 WT, n=9 KO). For subcutaneous and scapular adipose weights 16 weeks (n=7 WT, n=8 KO); 52 weeks (n=8 WT, n=10 KO). For gonadal adipose weights 16 weeks (n=7 WT, n=8 KO).


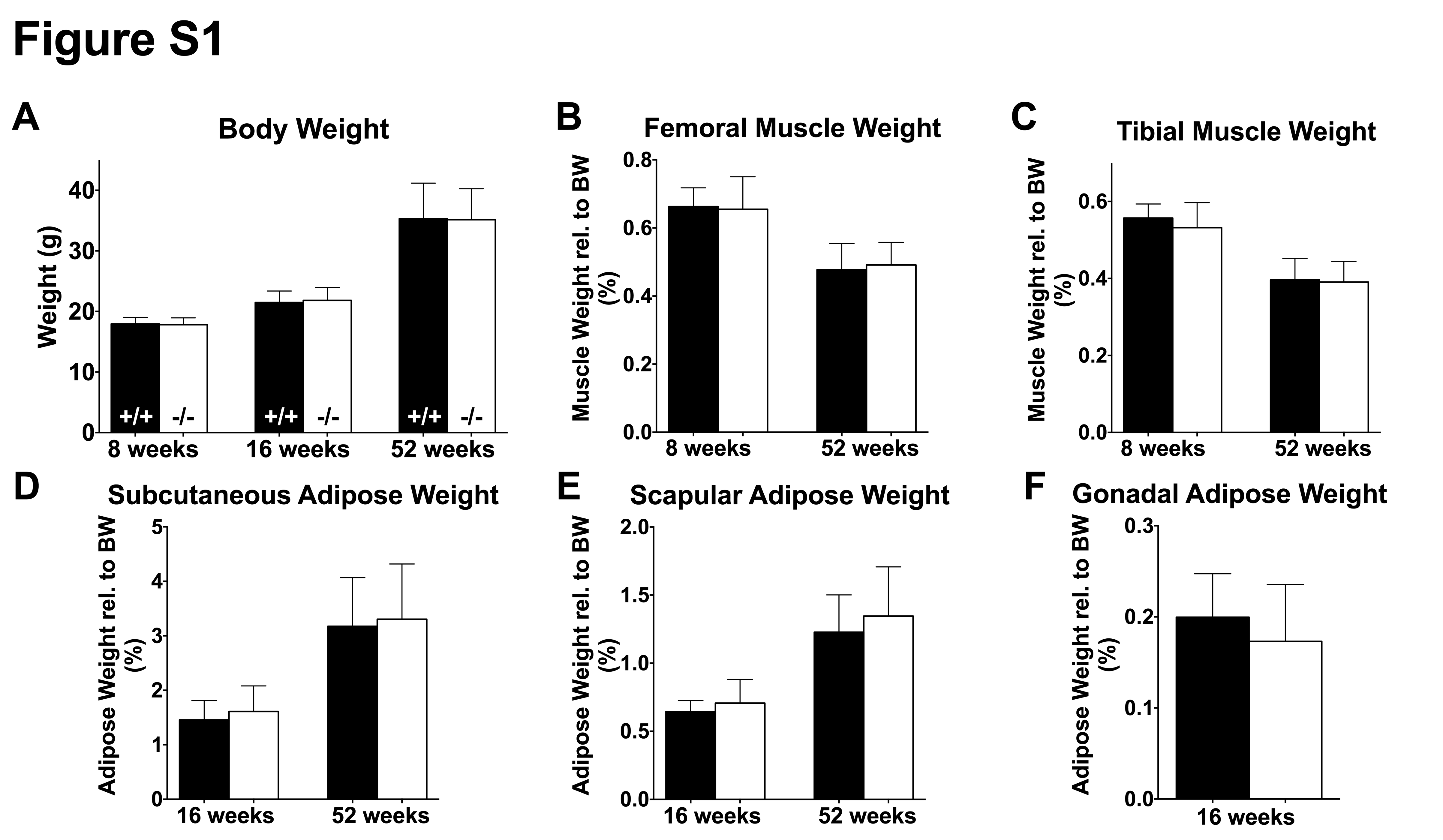


Figure S2: Body weight, femur length, and *ex vivo* evaluation of microarchitecture of femoral bones from 8-, 16-, and 52-week-old WT (+/+, black bar) and *Muc1* deficient (-/-, white bar) male mice. Total body weight of WT and KO male mice (A). Femur length of WT and KO male mice (B). Trabecular bone fraction (C), trabecular number (D), trabecular thickness (E), and trabecular separation (F) were measured at the metaphyseal region. Cortical bone area (G), marrow area (H), cortical thickness (I), and periosteal perimeter (mid-shaft circumference) (J) were measured at diaphyseal areas. For body weight 8 weeks (n=10); 16 weeks (n=9); 52-weeks (n=10 WT, n=9 KO). For bone length 8 weeks (n=9 WT, n=10 KO); 16 weeks (n=9 WT, n=8 KO); 52-weeks (n=8 WT, n=7 KO). For trabecular and cortical bone analysis 8 weeks (n=10); 16 weeks (n=9); 52-weeks (n=10 WT, n=8 KO). Statistics: student t-test exact p value, * = p<0.05 WT vs. *Muc1*^-/-^ within time point, and ** = p<0.01 WT vs. *Muc1*^-/-^ within time point. # = p < 0.05 compared to 8wk WT mice , ## = p < 0.01 compared to 8wk WT mice, ### = p < 0.001 compared to 8wk WT mice. $ = p < 0.05 compared to 8wk KO mice, $$ = p < 0.01 compared to 8wk KO mice, $$$ = p < 0.001 compared to 8wk KO mice.


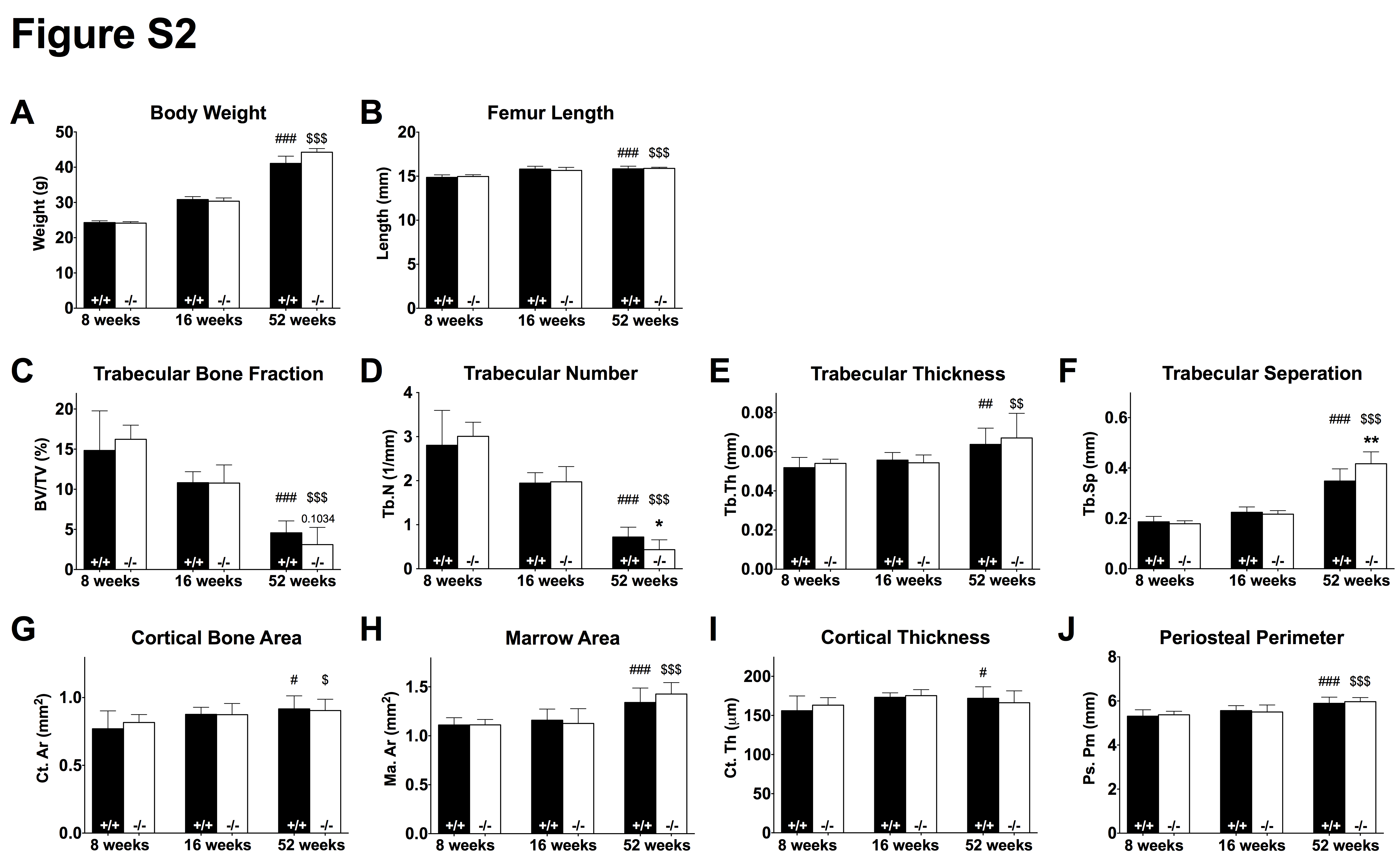


Figure S3: Mechanical testing of femurs of 8-, 16-, and 52-week old WT (+/+, black bar) and *Muc1* deficient (-/-, white bar) male mice. Three-point bending tests performed on the femurs of WT and KO mice allowed for quantification of the energy to failure (A), ultimate load (B), and bone stiffness (C) of the bones. Statistics: student t-test * = p<0.05 WT vs. *Muc1*^-/-^ within time point. ### = p < 0.001 compared to 8wk WT mice. $ = p < 0.05 compared to 8wk KO mice. $$ = p < 0.01 compared to 8wk KO mice. 8 weeks (n=10 WT, n=9 KO); 16 weeks (n=9 WT, n=10 KO); 52-weeks (n=10 WT, n=9 KO).


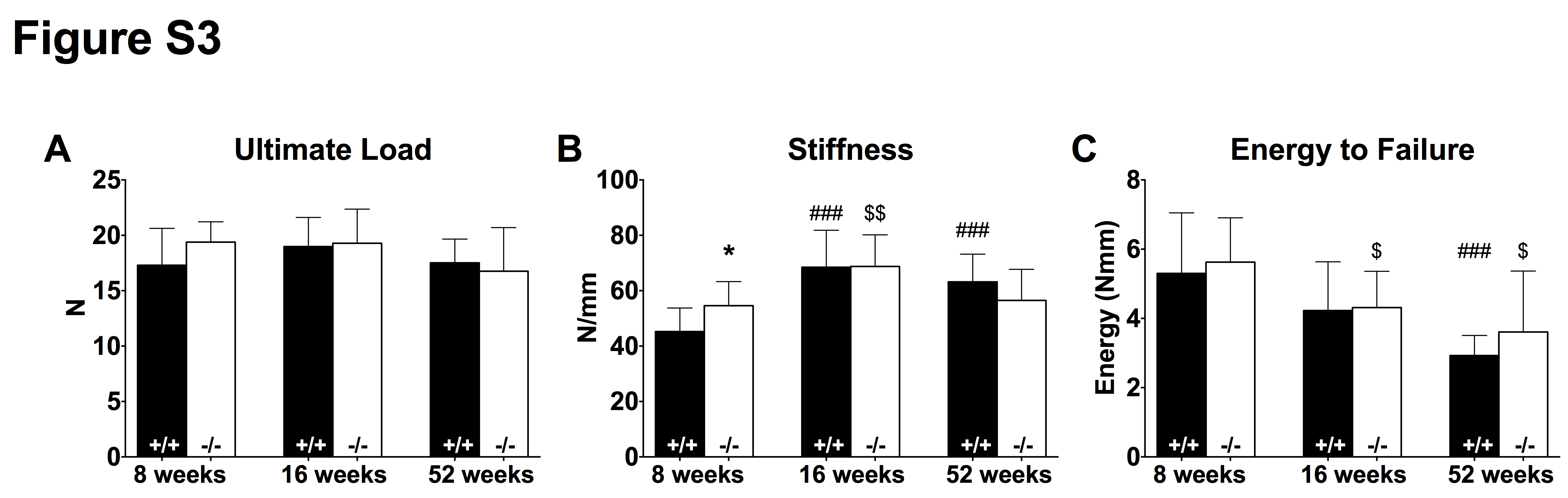


Figure S4: mRNA expression levels of *Muc1* in differentiation murine osteoclasts at day 3 (black bar) and 7 (white bar) as assessed by quantitative PCR. Results are presented as relative to the housekeeping gene *Hrpt*. n = 3 donor mice.


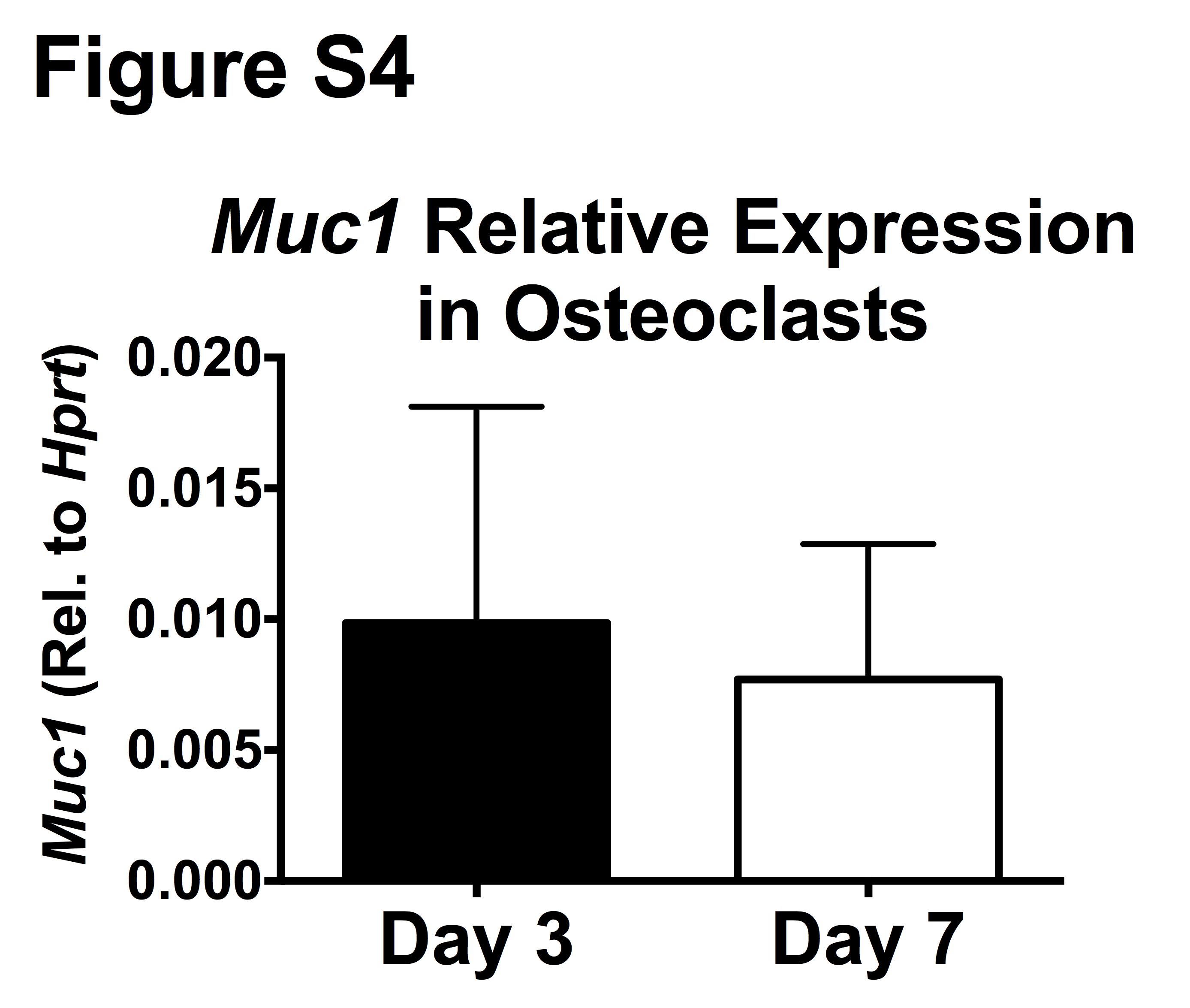

Supplement: Supplementary file 1 — Supporting Data S1. [file JBM4-2-341-s001.docx]
